# Design Principles for Pluripotent Stem Cell-Derived Organoid Engineering

**DOI:** 10.1155/2019/4508470

**Published:** 2019-04-18

**Authors:** Teresa P. Silva, João P. Cotovio, Evguenia Bekman, Maria Carmo-Fonseca, Joaquim M. S. Cabral, Tiago G. Fernandes

**Affiliations:** ^1^Department of Bioengineering and iBB-Institute for Bioengineering and Biosciences, Instituto Superior Técnico, Universidade de Lisboa, Av. Rovisco Pais, 1049-001 Lisboa, Portugal; ^2^The Discoveries Centre for Regenerative and Precision Medicine, Lisbon Campus, Universidade de Lisboa, Lisboa, Portugal; ^3^Instituto de Medicina Molecular, Faculdade de Medicina, Universidade de Lisboa, Av Prof Egas Moniz, Edificio Egas Moniz, 1649-028 Lisboa, Portugal

## Abstract

Human morphogenesis is a complex process involving distinct microenvironmental and physical signals that are manipulated in space and time to give rise to complex tissues and organs. Advances in pluripotent stem cell (PSC) technology have promoted the *in vitro* recreation of processes involved in human morphogenesis. The development of organoids from human PSCs represents one reliable source for modeling a large spectrum of human disorders, as well as a promising approach for drug screening and toxicological tests. Based on the “self-organization” capacity of stem cells, different PSC-derived organoids have been created; however, considerable differences between *in vitro*-generated PSC-derived organoids and their *in vivo* counterparts have been reported. Advances in the bioengineering field have allowed the manipulation of different components, including cellular and noncellular factors, to better mimic the *in vivo* microenvironment. In this review, we focus on different examples of bioengineering approaches used to promote the self-organization of stem cells, including assembly, patterning, and morphogenesis *in vitro*, contributing to tissue-like structure formation.

## 1. Introduction

The application of the biomimicry concept, defined as the imitation of biological systems, has contributed to a significant innovation in regenerative medicine during the last years. This concept is usually associated with new approaches that aim to achieve the recapitulation of the natural form or function, natural processes, or natural systems [1, 2]. In the bioengineering field, efforts have been made to mimic the natural forms and functions of the human body *in vitro*, from the molecular to the cellular level, in an attempt to recreate the highest complexity level, the organism.

Recently, with the discovery of the ability of pluripotent stem cells (PSCs) to coordinate various key signals and to recapitulate different structures as seen *in vivo*, including tissue- and mini organ-like structures, our knowledge about human development and morphogenesis in healthy and disease contexts has been greatly improved [3, 4]. With the recapitulation of human organogenesis *in vitro*, the concept “organoid” emerged. In 1946, the “organoid” term was employed for the first time to define a tumor-derived mass isolated from a human tissue [5]. Subsequently, all tissue masses resultant from transplants were defined as “organoids” [6, 7], and the concept evolved to include cultures that were generated from dissociation and aggregation of animal- and tissue-derived cells [8–10]. With the recent advances in human PSC expansion culture and direct differentiation, the “organoid” definition followed the same evolution, nowadays referring to an *in vitro* 3D multicellular structure containing different cell types with self-organization, as seen in human tissues, typically derived from stem cells [2]. Frequently, organoids display spherical or irregular shapes in suspension or are embedded in different types of matrices [11].

The recreation of functional and structural mimicry within the organoid requires a minimal number of design components inspired on the original biological system. These include cellular and noncellular parameters, such as cell type and microenvironmental and physical parameters, as well as the resulting internal and external interactions, like cell-cell, cell-matrix, and cell-microenvironment [12]. The ultimate goal is to reestablish some of the features of human tissues, particularly the presence of different cell types to recapitulate the multicellular heterogeneity, and to control the microenvironment to recreate a high level of organization, promoting organoid maturation to achieve tissue functionality [11]. Thus, the application of bioengineering strategies to manipulate cellular and noncellular components may become a powerful tool to direct 3D human organoid morphogenesis.

The remarkable progress in organoid generation has provided the possibility to use these novel platforms for understanding human development and the complex processes involved in organogenesis. The use of organoids in drug screening and toxicological testing could also improve the safety and efficiency of drugs before reaching clinical trials, making the drug development process more cost-effective. Lastly, disease-derived organoids could also offer a valuable platform to study the mechanisms involved in disease manifestation and to identify possible therapeutic targets.

Here, we review distinct bioengineering approaches to direct the stem cell commitment and further self-organization of cells, recapitulating tissue morphogenesis *in vitro*. First, the self-organization capacity of cells is explored based on cell-cell and cell-matrix interactions. Afterwards, as self-organization is based on three different cell-associated capacities, including self-assembly, self-patterning, and self-morphogenesis, we highlight examples of bioengineering methodologies to control the initial state and the spatiotemporal positioning of cells and, lastly, the growth and remodeling of multicellular aggregates to achieve complex structures (Table 1).

## 2. Self-Organization in PSC-Derived Organoids

**Figure 1:**
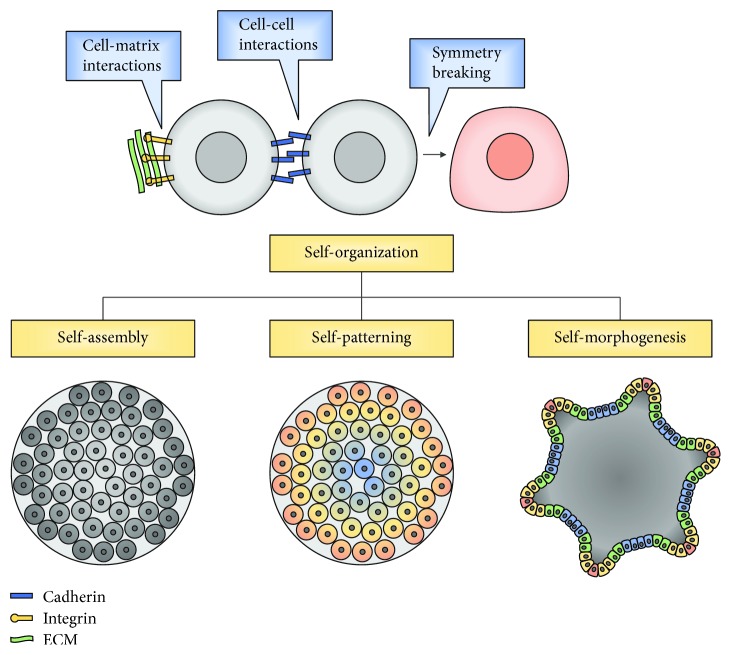
In tissue morphogenesis, the self-organization capacity of cells is achieved by a multicellular process involving cell-cell and cell-matrix interactions, as well as symmetry breaking. This capacity includes a combination of self-assembly, self-patterning, and self-morphogenesis capacities, which involves the control of the cell position, spatiotemporal control of cell stage, and control of tissue mechanics.

The ability of human PSCs to produce highly organized structures that reproduce features similar to the embryo and adult tissues was first detected in the teratomas formed after the injection of human embryonic stem cells (ESCs) in immune-deficient mice (reviewed in [13]). The “self-organization” capacity involves three different categories: first, the control of relative cell position, named “self-assembly”; second, the spatiotemporal control of the cell stage, defined as “self-patterning”; and lastly, the capacity to promote deformation, growth, and remodeling, which is termed “self-morphogenesis” (Figure 1) [14]. This intrinsic ability of organization is strongly dependent of the physical and morphological properties of cells, the autologous and exogenous signals that they receive, and also the mechanical features of the system.

### 2.1. Cell-Cell Adhesive Interactions

During embryogenesis, cell-cell interactions play a critical role in the dynamic changes of cell sorting, arrangement, and migration that originate different tissue morphologies. The adhesive forces between cells are crucial for the assembly and organization into a 3D structure. The most important and global mechanism of cell adhesive interactions is mediated by cadherins, which are Ca^2+^-dependent transmembrane proteins that facilitate homophilic cell-cell adhesion by their extracellular domains, whereas the intracellular domain interacts with their partner proteins, the catenins (reviewed in [15]). Following cell-cell adhesion, a protein complex is formed composed by the catenin polypeptide of *α*-, *β*- or *γ*-catenins (reviewed in [16]). Subsequently, *α*-catenin mediates physical interaction to the actin cytoskeleton, demonstrating that cadherins can also guide cell cytoskeletal anchoring [17, 18]. Different cadherins are expressed in different tissues, and the best-studied are the classical vertebrate cadherins, including N-cadherin, highly expressed in the neuronal tissue [19, 20], and E-cadherin, mostly expressed in epithelial cells [21]. Nonclassical cadherins can be found in other human tissues, for instance, VE-cadherin, which is the vascular-endothelial cadherin [22], and R-cadherin, expressed in the retinal tissue [19].

**Figure 2:**
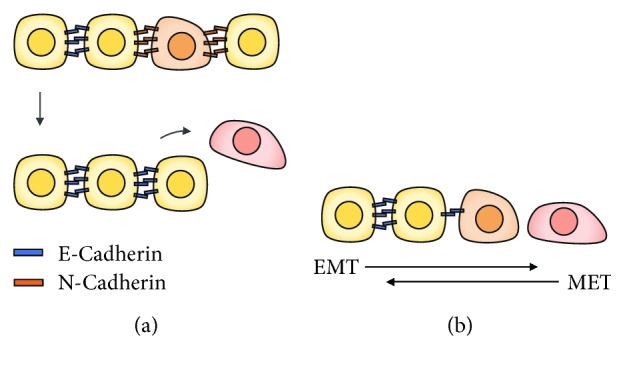
Cadherin involvement in tissue morphogenesis. (a) Cell sorting based on differential expression of distinct cadherins. (b) Epithelial-mesenchymal transition (EMC) and its reverse (MET), an example of self-assembly capacity mediated by differential cadherin expression and regulation.

During morphogenesis, different mechanisms involving cadherins appear to influence cell sorting and therefore alter the spatial organization of cells. The expression of different types of cadherins in different cell types promotes the selective recognition and connection of cells expressing the same type of cadherin leading to cell sorting and separation into different tissues [23–25]. For instance, N-cadherin expression in neural cells allows the separation from epithelial cells that express E-cadherin (Figure 2(a)) [26]. In other cases, independently of the cadherin type expression, cell sorting is also observed based on differential levels of cadherin expression [25, 27, 28]. The epithelial-mesenchymal transition (EMT), the reverse of epithelization, is a strong example of the self-assembly capacity of cells mediated by cadherin expression and regulation (Figure 2(b)) [29]. This process is achieved by altering cell-cell contact and promoting cell migration. In particular, E-cadherin is downregulated during the transition to the mesenchymal state, leading to the decrease of cell-cell interactions [30, 31]. Simultaneously, an alteration on cellular signaling profiles and a remodulation of the cytoskeleton is observed, allowing cell migration (reviewed in [32]).

In addition, other physiological factors that interact with cadherin-mediated signaling can influence cell sorting independently of cadherin expression. During development, an anterior-posterior axis is created leading to the formation of compartment boundaries. Although epithelial cells express high levels of E-cadherin, selective adhesion is observed creating different boundaries in response to Hedgehog (Hh) signaling [33, 34]. Activation of Hh expression in posterior cells conduces to diffusion of signals across the anterior-posterior boundary that determine the sorting of some anterior cells next to the boundary, which are not capable of receiving Hh and are sorted toward the posterior region [34]. Besides that, the dynamic regulation of cadherin adhesions may drive cell rearrangements and migration. By breaking and reforming cadherin adhesive bonds, the convergent extension movements contribute to tissue morphogenesis by changing the local cellular arrangement with respect to neighboring cells [35, 36].

Besides the important function of cadherins during morphogenesis, their critical role in cell aggregate formation and further differentiation was already demonstrated. By inhibition of E-cadherin-mediated adhesion, the agglomeration of ESCs in cell aggregates is prevented as well as their differentiation [37–39]. Hence, technologies to control stem cell differentiation by manipulating cell-cell interactions have been created. For example, surface engineering by immobilization of cadherins has been used to manipulate cadherin-mediated signaling pathways and thus direct stem cell fate decisions [40, 41]. Moreover, it was demonstrated that not only does the immobilization of cadherins mediate stem cell differentiation but the interaction with adjacent cells also has an important role in patterning particular cell types. The incorporation of certain progenitor cells allows the addition of specific cell-cell interactions that mimic *in vivo* conditions and manipulate differentiation processes. For example, coculture with organ-matched mesenchymal cells allows the proliferation of progenitor cells, without differentiation, giving rise to progenitors that were able to efficiently produce large numbers of specific differentiated cells [42].

### 2.2. Cell-Matrix Interactions

Not only do cell-cell interactions provide important signals in the cell niche but other structural, physical, electrical, or biochemical signals present in the complex microenvironment during embryonic development also affect cell fate decisions (reviewed in [43]). The extracellular matrix (ECM) is an important component that gives the structural support to the cell niche and also contributes for mediating signaling for cell migration, retention, and polarization [44, 45]. The ECM is composed primarily by glycosaminoglycans and fibrous proteins that are secreted by the cells to generate their own physical scaffold (reviewed in [43]). Cells interact with ECM molecules via integrins, which are cell adhesion receptors, regulating cellular behavior (reviewed in [46]).

Integrins present a family of heterodimeric transmembrane glycoproteins where heterodimers are composed of non-covalently connected *α* and *β* subunits [47]. In vertebrates, 24 different heterodimers resulting from different assemblies of 18 *α* subunits and 8 *β* subunits have been described. Based on their subunit composition, integrins can be classified in different subgroups. Under certain conditions, each cell type exhibits a specific integrin signature, including the subgroup and quantity of integrins (reviewed in [48]). However, this is a dynamic process, and both the developmental stage and microenvironmental conditions can change the integrin repertoire (reviewed in [49]). While the extracellular domain of integrins interacts with components of ECM, including fibronectin, laminin, and collagens, the intracellular domain links to cytoskeletal and regulatory proteins, such as *α*-actinin, filamin, calreticulin, and cytohesin (reviewed in [50]). It is also known that the same component of ECM interacts with different integrin receptors, and in the same way, a specific integrin receptor may recognize different ECM components (reviewed in [48]).

The role of integrins during embryogenesis has been extensively studied, and the data accumulated so far are already enough to place integrins as important players in fertilization, cell migration in gastrulation, adhesion in embryo implantation, and generation of different organ systems, like the nervous system (reviewed in [50]). Additionally, it was already shown that the composition of ECM is able to influence ESC behavior in the development of 3D structures as well as their differentiation. For example, fibronectin was reported to strongly stimulate endothelial and vascular cell differentiation, while laminin promotes the generation of beating cardiomyocytes [51]. The matrix that is most commonly used for PSC differentiation and generation of different types of organoids is Matrigel, which is a gelatinous protein mixture extracted from Engelbreth-Holm-Swarm mouse sarcoma cells [52, 53], prone to lot-to-lot variation. There are few studies that try to address the exact mechanism by which Matrigel supports organoid development. Although the manipulation of integrin signaling to direct stem cell fate is still very difficult, some groups have been studying the involvement of specific integrins in PSC differentiation, with a focus on identification of ECM components directly interacting with a specific integrin subgroup and promoting selective endoderm [54], mesoderm [55], or ectoderm [56] differentiation.

In addition to these chemical cues from the ECM, mechanical and physical stimuli, like porosity and stiffness, also exert their influence on cellular commitment [57]. The matrix stiffness can be sensed by cells through mechanoreceptors that also include integrins, regulating cellular behavior (reviewed in [58]). While intermediate substrate stiffness favors the endodermal lineage, softer substrates originate ectodermal tissues [59]. It was also demonstrated that mesodermal differentiation is very sensitive to mechanical properties of the ECM [60]. While soft substrates enhance mesoderm commitment, stiff matrices induce only minimal mesoderm differentiation [60]. In this latter study, authors showed that on a soft substrate, human ESCs present *β*-catenin accumulation at cell-cell adhesions leading to enhanced WNT signaling and subsequent WNT-dependent mesoderm differentiation. In contrast, stiff materials promote the integrin-dependent *β*-catenin degradation and thus inhibit mesoderm commitment [60]. Therefore, by playing with biochemical components of the ECM, as well as its mechanical and physical parameters, cell proliferation and differentiation can be manipulated in the 3D microenvironments.

### 2.3. Breaking Symmetry

**Figure 3:**
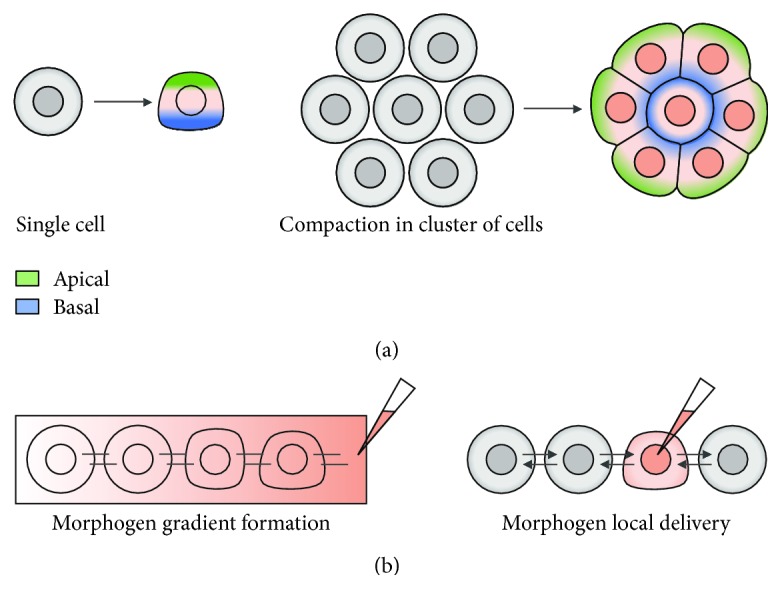
Symmetry breaking process. (a) Symmetry breaking *in vivo* is observed at the single-cell level and multicellular level, involving a process of compaction. (b) Different approaches for symmetry breaking *in vitro*, using microfluidic approaches to create a morphogen gradient or local delivery of morphogens.

Symmetry breaking is a pivotal phenomenon in animal development that precedes pattern formation, allowing the generation of higher morphological and functional specialization. *In vivo*, symmetry is broken at the single-cell level, where the cellular cytoskeleton and membrane-associated proteins are redistributed to create apicobasal polarity (Figure 3(a)). For example, while integrins accumulate at the basal side of the cell, a ring of actin filaments is formed at the apical side. The actin ring contraction can drive apical constriction leading to cell shape alteration and epithelial sheet bending (reviewed in [61, 62]). In addition, symmetry breaking also occurs at the multicellular level, as seen in the early mouse embryo. This morphological event called compaction transforms the embryo from a loose cluster of spherical nonpolarized cells into a tightly packed mass, in which cell-cell contacts are strengthened and cell polarization is achieved (Figure 3(a)). Several mechanisms are involved in the compaction process: cell-cell adhesive interactions, involving the redistribution of E-cadherin; cortical tension, generated by actomyosin network contractility determining the cell shape; and extension of long membrane protrusions (reviewed in [63, 64]).

The precise molecular and physical features, as well as the precise timing in which symmetry breaking occurs, are still poorly understood. Some events appear to be cell-autonomous, depending on the asymmetric gene expression in embryonic cells, and others appear to be caused by morphogen gradients. In fact, symmetry breaking can be achieved by an initially homogeneous morphogen distribution, which can turn into a concentration gradient due to reaction-diffusion [65]. In a reaction-diffusion model, the self-organization capacity of cells leads to symmetry breaking activated by a stochastic disturbance of the system without a requirement of a dominant “master factor” [66]. Therefore, cell characteristics, including gene expression and cell polarity, and local interactions between cells can by themselves be responsible for lineage establishment. Reported studies already demonstrated that a uniform aggregate of stem cells is capable to originate a high level of organization, comparable to what is observed in native tissues [67–69]. Some organoid models with minimal but sufficient complexity are able to undergo spontaneous symmetry breaking in the absence of spatial cues. In this case, a specific pattern is created including rostral-caudal polarization in cortical organoids [67], anterior-posterior patterning in 3D gastruloids [68, 70, 71], and dorsal-ventral patterning in neural tube organoids [69, 72]. Therefore, symmetry breaking events can be attained *in vitro* by the addition of a single morphogen, through a diffusion-reaction mechanism, or by using more sophisticated bioengineering approaches to create symmetry breaking based on local morphogen delivery (Figure 3(b)).

## 3. Controlled Assembly of PSCs

The generation of organoids starts by promoting the assembly of PSCs into a 3D structure. Similar to the human embryo, the earliest cell fate decision is based on the spatial orientation of cells (reviewed in [64]). Therefore, methodologies to control cell arrangement during the initial organoid assembly can affect further morphogenesis induction. The assembly process can be achieved based on the self-assembly properties of cells in a scaffold-free tissue engineering approach or by using different bioengineering strategies to direct and control the arrangement of cells.

### 3.1. Scaffold-Free Approaches

The generation of organoids in a scaffold-free manner is based on the “self-organization” property of stem cells, in which cells have the ability to assemble in a 3D structure. Different methodologies have been applied to form 3D aggregates of PSCs, with the embryoid body (EB) formation by the hanging drop method the first to be used for the production of homogeneous cell aggregates. This technique is based on gravity to force the cells to aggregate and consists of creating small drops of a medium with cells suspended on a lid [73]. To overcome the manipulation limitations that could disturb the EBs, this technique was adapted to V-bottomed and round-bottomed multiwell plates, in which cells are forced to rapidly aggregate by applying a rotational force [74]. However, this methodology does not avoid the individual manipulation of the cell aggregates. Therefore, different microwells fabricated by lithographic techniques have been used to simultaneously generate 100s to 1000s of cell aggregates by centrifugation, allowing the scaling up of the multiwell plate technique [75–77]. In addition, microfluidic channels have also been used for the continuous formation of cell aggregates, being a powerful tool for high-throughput applications [78].

In these scaffold-free methodologies, the most important parameter to be controlled is the size of the generated aggregates. It was demonstrated that the size of the cell niche influences the differentiation trajectories because of its impact on the microenvironmental parameters, including the spatial gradient of soluble molecules, and cell-cell and cell-matrix interactions [79, 80]. Thus, since variations in cell number are translated to different aggregate sizes, controlling the cell aggregate size can influence the signaling pathways conducing to a more efficient commitment and differentiation. In fact, different research groups have been optimizing the aggregate diameter to improve the mesoderm or neuroectodermal induction, achieving higher yields of cardiac and neuronal cells [81–83].

More recently, Xie et al. reported that not only the size of cell aggregates can influence the differentiation toward different lineages but also the self-assembly kinetics. The study showed that the aggregation kinetics altered the EB structure; in particular, slower kinetics originated EBs with higher porosity facilitating the exposure of cells to growth factors. Ultimately, faster aggregation appears to favor ectodermal commitment whereas slower aggregation promotes mesoendodermal differentiation [84].

### 3.2. Scaffolds for Imposing External and Internal Architecture

Cellular organization within an engineering tissue involves the assembly of cells into a specific arrangement for mimicking the architecture of the native tissue. To mimic the *in vivo* physical and biochemical properties of the tissue microenvironment, different matrices can be used, including those from natural sources or artificially synthesized. A specific architecture can be externally imposed by using different approaches to manipulate the tissue shape, like molds and scaffolds. For example, microcontact printing can provide different molds from different materials like agarose, polydimethylsiloxane, or polyacrylamide, with minimal adhesive properties, only to force cells to aggregate and acquire a specific shape [85, 86]. Besides that, this technique can be used to introduce some functionalization by directly depositing proteins or ECM components onto a partially polymerized substrate [87]. Furthermore, the control of the shape, size, space, and organizational symmetry of nanometer-scale features in different biomaterials has been achieved by using different nanolithography strategies. Among different nanotopography approaches, the electrospinning allows the formation of nanofibrous substrates from natural or synthetic polymers, while electron-beam, selective etching, and nanoimprinting have been used to create nanopits, nanopillars, or nanochannels on various materials. By applying these different approaches, the natural dimensions of basement-membrane fibers and pore sizes can be reached allowing to mimic the porosity of the natural ECM (reviewed in [88]).

The scaffolds used for imposing the external shape and mimicking the natural ECM mostly have a fixed morphology. However, human development starts on a microscale, and considerable morphologic changes have to occur to achieve the final morphogenesis. Therefore, it is very important to try to dynamically control the organoid morphology in order to reach a correct tissue-like organization. The application of different types of hydrogels has been able to improve the control of the 3D microscale morphology of organoids. Hydrogels are hydrophilic 3D polymeric networks with natural or synthetic origin that are insoluble due to the presence of chemical or physical crosslinks [89, 90]. The internal structure of the hydrogel can be manipulated by using different techniques, including 3D printing [91] and sacrificial molding [92], which can possibly regulate the morphology of the generated structures.

In the last years, significant improvements have been made concerning mechanical performance and functionality in the 3D printing of hydrogels. There are different reported hydrogel composite 3D printing techniques that allow to fabricate complex and highly customizable scaffold structures, including nozzle-based, laser-based, and inkjet-based 3D printing systems (reviewed in [93]). The nozzle-based 3D printing is the most used approach, in which viscous liquids or melted polymers are forced to extrude out of a nozzle, syringe, or orifice in order to sequentially build a 3D structure based on a predesigned path created by computer modeling. Recently, Hinton et al. reported an adaptable and cost-effective nozzle-based 3D printing, termed freeform reversible embedding of suspended hydrogels (FRESH), that uses a thermo-reversible support bath to enable deposition of hydrogels. Based on 3D imaging data from whole organs, FRESH is able to print scaffolds with complex internal and external architectures, including a 3D CAD model of the embryonic heart [94], demonstrating a valuable applicability in organogenesis. In addition, the laser-based 3D printing systems are also capable of building 3D structures in photo-treatable hydrogels under the deposition of laser energy, normally UV light, into specific designed patterns [95]. Finally, inkjet printing is a noncontact printing technique used to create ink droplets onto a material platform (reviewed in [96]). Even though biological molecules and structures are fragile and sensitive, this approach appears to be appropriate to introduce biological modification on generated scaffolds, since it already was successfully used to transfer biomolecules like nucleic acids to solid supports [97].

Miller et al. were the first to report the generation of cylindrical networks within different hydrogels by using 3D filament networks of carbohydrate glass as a sacrificial template [92]. Therefore, they were able to pattern vascular networks into 3D tissue constructs by molding channels. Following this study, this sacrificial molding technique was also used by other groups to create internal cavities of micro- to macroscale dimensions within a variety of hydrogel materials by applying different molds, including calcium alginate and polyvinyl alcohol (PVOH) templates [98, 99]. Briefly, the sacrificial molding technique is based on encapsulating a dissolvable or degradable material within a second hydrogel material. After the composite hydrogel formation, the internal sacrificial material is removed and a hydrogel with defined internal architecture is created. This internal architecture manipulation in the hydrogels provides an important tool not only to create vascularized tissues but also for organoid encapsulation, in which the internal spaces allow the growth, deformation, and remodeling of the organoids to generate a defined morphology.

### 3.3. Bioengineering Approaches to Manipulate Organoid Assembly

Several bioengineering approaches have been applied to guide cell assembly in order to achieve a desired cell arrangement and organoid shape. In 2015, Todhunter et al. reported a DNA-programmed assembly of cells (DPAC), in which size, shape, composition, and spatial heterogeneity is programmed, thereby recreating the multicellular organization of organoids [100]. In DPAC, 2D DNA-patterned substrates are used to guide cellular organization by presenting complimentary lipid-modified oligonucleotides. After this programmed assembly, a DNase treatment is performed to release a well-organized cell aggregate, followed by encapsulation within ECM gels [100, 101].

As previously described for fabrication of scaffold structures, 3D printing techniques have also been applied to control cell assembly by the deposition of single or multiple types of cells with different supportive matrices. This type of bioprinting methodology involves different approaches, like inkjet bioprinting, microextrusion systems, and laser-based direct-write techniques, in which different actuation methods are applied (reviewed in [102]). In inkjet bioprinting, two different actuation methods are used, piezoelectric and thermal, whereby either acoustic waves or thermal forces, respectively, are used to prepare liquid droplets. While in the thermal approach a variable size of droplets is obtained, in the piezoelectric technology, regular and equal sizes are generated [103, 104]. This is a low-cost technology with high resolution and printing speed; however, it has some limitations regarding the type of materials that can be printed. Although some thermal and mechanical stress can be introduced to the cells, this technology was already successfully applied to different mammalian cell printing with a viability above 85% [104]. On the other hand, the microextrusion technique is derived from the modification of inkjet printers, which are pressure-assisted robotic apparatus that can extrude cell-laden hydrogels by pneumatic or mechanical dispensing onto a substrate (reviewed in [105, 106]). Human chondrocytes and osteogenic progenitors in combination with an alginate hydrogel were already extruded by using a pneumatic syringe dispenser, demonstrating the ability to create 3D structures with high cell viability [107].

The laser-based direct-write technique is the most applied bioprinting technology. This technique uses a laser beam that is focused on a support layer underlying a cell-containing matrix on the donor print ribbon, forcing its rapid volatilization and allowing the cell to be transferred onto an adjacently localized receiving substrate (reviewed in [108]). High cell viabilities have been reported using this technique, due to low shear stress, and its high resolution allows single-cell deposition, yielding scaffold-free 3D cell constructs [109]. For cell-based applications, the most common laser-based techniques are biological laser processing (BioLP), matrix-assisted pulsed laser evaporation direct writing (MAPLE-DW), laser-induced forward transfer (LIFT), absorbing film-assisted laser-induced forward transfer (AFALIFT), and laser-guided direct writing (LG-DW) (reviewed in [108]). MAPLE-DW was successfully used to deposit patterns of different cell types onto and within the Matrigel, demonstrating that spatial coherence can be achieved [110, 111]. Furthermore, human osteosarcoma cells were printed by BioLP and transferred into the Matrigel, producing a 3D cellular construct with 95% of cell viability [112]. This method was later improved reaching 100% viable cells and single-cell resolution [113]. Thus, a high control in cell assembly is reached, allowing to manipulate cellular arrangement and composition within an organoid with a defined 3D microscale morphology.

## 4. Directed Organoid Patterning and Morphogenesis

**Figure 4:**
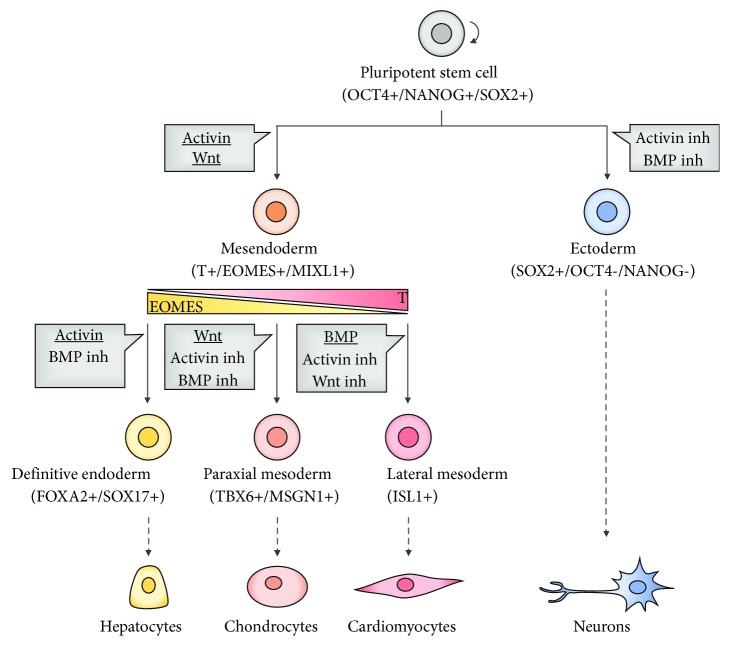
Lineage specification from PSCs. Ectoderm induction is achieved by dual SMAD inhibition, whereas mesendodermal differentiation is based on the activation of dual SMAD regulators and WNT signaling. Dashed arrows represent examples of different cell types achieved by manipulating specific lineage differentiation.

The knowledge about the signaling pathways involved in pluripotency maintenance, as well as the generation of different germ layers, has allowed the manipulation and control of PSC commitment to different lineages and further differentiation into specific cell types. For instance, neuroectoderm commitment is easily achieved by manipulating TGF*β* signaling [114]. The most efficient method for neural induction from PSCs is the dual SMAD inhibition of BMP and activin signaling (Figure 4), which are antagonized by Noggin and Lefty, respectively [114, 115]. Other chemical antagonists have been used to promote BMP signaling inhibition, like Dorsomorphin and LDN-193189, blocking the commitment towards the trophectoderm [115, 116]. For nodal/activin signaling inhibition, a chemical antagonist SB431542 is efficient to prevent the mesendodermal differentiation by blocking the TGF*β* signaling [115, 117].

Oppositely, the activation of dual SMAD regulators, as well as the WNT signaling, appears to be critical for the initial mesendoderm commitment, giving rise to Brachyury^+^ (T)/EOMES^+^/MIXL1^+^ cells [118, 119]. Following mesendoderm induction, by manipulating the levels of T and EOMES, further differentiation towards the mesoderm or endoderm can be specified (Figure 4) [120]. T action seems to be important for the mesodermal fate, repressing endodermal differentiation [120]. On the other hand, high levels of EOMES are essential for the expression of endoderm markers (FOXA2 and SOX17) [121]. While activin leads to the development of a population with higher expression of FOXA2, resulting in endoderm specification, WNT activation generates cells with lower expression of FOXA2, important for the mesoderm fate [122]. Interestingly, although BMP is not required for the mesendoderm commitment, alone, it is capable of inducing the development of a population with low expression of FOXA2 [119, 123]. In mesodermal specification, WNT and BMP signals induce bifurcation of two mesoderm subtypes, the paraxial and the lateral mesoderm, respectively [124]. While WNT appears to be important for mesoderm specification and further generation of chondrocytes, the inhibition of WNT signaling is essential to promote cardiac differentiation [124–126].

On the other hand, after the establishment of the activin-induced definitive endoderm, various cell populations can arise, such as hepatocytes and pancreatic cells. BMP and WNT signaling pathways have an important role in the generation of the pancreatic lineage, while the specification of insulin-producing cells can be achieved by FGF signaling [127, 128]. The combination of FGF and BMP4 signaling is related with hepatic fate specification [129].

Based on the manipulation of the previously described signaling pathways, as well as on the “self-organization” capacity of stem cells, organoids from different lineages have been produced including the brain, kidney, liver, pancreas, lung, and gut [3, 130–134]. Eiraku et al. were the first to demonstrate the ability of PSCs to self-organize in the cortical tissue and recapitulate embryonic brain development [135]. Later, Lancaster et al. were able to direct human PSC differentiation into different cerebral cortex regions and apply this technique for disease modeling [3]. A variety of well-organized neuronal organoids was later reported, including forebrain-, midbrain-, hypothalamus-, and cerebellum-like structures [136–139]. In addition to brain organoids, by directing PSCs towards the intermediate mesoderm, organoids that recapitulate the first trimester of the human fetal kidney were also generated. These organoids present individual nephron-like structures segmented into distal and proximal tubules surrounded by endothelia and renal interstitium, demonstrating a well-organized structure [133]. These are some examples of the ability to recapitulate human organogenesis *in vitro* from PSCs by the addition of only a few signaling cues. However, differences between native organ- and PSC-derived organoids can still be observed. This can result from inappropriate selection of microenvironmental cues or static signaling presentation in both space and time. Therefore, a higher spatiotemporal control is required to achieve closer similarity to the native microenvironment.

### 4.1. Bioengineering Approaches for Spatiotemporal Control of Mechanical Signals

As previously demonstrated, the mechanical properties of the cellular microenvironment strongly influence cell differentiation, as well as cellular proliferation and apoptosis [60, 140, 141]. Additionally, such mechanical features are specific for different organs, or even within the same organ, different components present distinct mechanical properties, allowing the modulation of cellular behavior and promoting multicomponent organogenesis [142, 143]. Subsequently, for organoid generation, the spatial modulation of mechanical features is a critical issue that can be achieved by generating composite hydrogels. For instance, the functionalization of traditional hydrogels, such as the Matrigel or collagen, with synthetic ECM analogs allows to manipulate the mechanical properties [144, 145]. The incorporation of adhesion peptides permits to manipulate mobility, since long peptide tethers lead to cell attachment and spreading, whereas short peptide tethers induce cell adhesion resistance, resulting in cell clustering [146]. Also, the incorporation of peptide substrates that are susceptible to enzymatic cleavage can also modulate hydrogel degradation by cells and therefore promote cell migration [147]. A modular design of silk protein-based porous scaffolds was also used to produce combined hydrogels, recreating the six-layered architecture of the human cortex. The reported approach consisted of an adhesive-free assembly of concentric units to create the modular structures based on a jigsaw puzzle-like cutting process. In this way, different layers were populated with distinct types of neurons, and a functional 3D cortical tissue construction was reached [148]. An alternative route to produce complex structures with composite hydrogels is a DNA-directed assembly of shape-controlled units. This technique presents the same principle as previously described to cell assembly, consisting in the enrichment of different blocks with circle DNA strand “glues.” Based on the complementarity of each DNA block, a programmable assembly of complex macroscale structures can be achieved [149].

In addition to the reported technologies that allow the spatial modulation of mechanical features, further temporal guidance is possible to be generated by light-mediated patterning. The formation of photodegradable hydrogels by incorporating photolabile moieties within the network backbone of a hydrogel, like poly(ethylene glycol), makes the manipulation of the physical features of the ECM possible by using the light of different wavelengths. Upon light exposure, the local network crosslink density decreases and the hydrogel is cleaved, resulting in changes in physical properties, including stiffness, water content, diffusivity, or complete erosion, even in the presence of cells [150]. In contrast to this local softening, the presence of a photoinitiator originates additional crosslinking after the exposure to ultraviolet light, providing local stiffening [151].

In addition to light-patterning, other approaches have been applied for tuning the stiffness of hydrogels by using a combination of a pH-sensitive poly(2-(diisopropylamino)ethyl methacrylate) (PDPA) and biocompatible poly(2-(methacryloyloxy)ethyl phosphorylcholine) (PMPC) [152]. With the careful adjustment of the pH, the hydrogel film elasticity could be reversibly modulated allowing for the stiffening or softening of the material and for the temporal dynamic manipulation of cell adhesion/detachment [152]. This reversibly tunable stiffness can also be reached in cell-laden hydrogels based on supramolecular “host-guest” interactions. In this reported method, the stiffness is manipulated by noncovalent and reversible host-guest interactions between pendant “host” motifs, which are present in the primary hydrogel network and soluble polymers. Thus, when these soluble polymers are added, additional physical crosslinks are formed resulting in increased hydrogel crosslinking density and elastic modulus [153]. Hydrogels can also be dynamically stiffened by using enzymatic reactions, in which a peptide linker with additional amino acid residues that are susceptible to a specific enzyme catalyzation is created. After enzyme exposure, a specific dimer formation is achieved leading to additional crosslinks and final stiffening of the cell-laden hydrogel [154].

Therefore, based on these techniques, a spatiotemporal patterning of the mechanical features is straightforwardly reached. And by manipulating the matrix stiffness, the growth of neighboring tissues and consequently the mechanical confinement as seen *in vivo* could be mimicked in the organoid microenvironment [155].

### 4.2. Bioengineering Approaches for Spatiotemporal Control of Morphogen Diffusion

Morphogens are molecules that are able to coordinate organ growth and patterning, establishing a graded concentration distribution and eliciting distinct cellular responses in a dose-dependent manner. They can be either cytoplasmatic proteins, able to promote a diffusion gradient within the cell, or secreted signaling molecules [156]. The gradient of these signaling molecules appears to direct tissue patterning during embryogenesis [157, 158]. The formation of specific structures can be induced by gradients of signaling molecules produced by the neighboring cells and leading to differential gene expression, tissue patterning, and morphogenesis [159]. *In vitro*, various morphogens, including small molecules, growth factors, and hormones, have been used to regulate cell fate within the organoids. Furthermore, advances in the bioengineering field allowed for the spatiotemporal control of the morphogen gradients within the organoid making possible to instruct the correct morphogenesis.

As already mentioned above, light-mediated patterning approaches present also a promising application to control morphogenic signals in both space and time. Biomolecules can be introduced within the hydrogel at a desired location, protected by a photodegradable moiety [160, 161]. At the proper timing, light exposure leads to a specific photo-releasing of the biomolecule. Beyond the spatial and temporal delivery control, for a given light exposure, the amount of biomolecule that is released can be predicted [161]. Additionally, the use of microfluidic systems or micro/nanoparticles allows an efficient spatiotemporal control of morphogen gradients. Lithographic techniques can be used for the production of channels to create functional microfluidic structures within hydrogels. Given the hydrodynamic properties of microchannels, following the initial homogeneous distribution of biomolecules inside the channels, a concentration gradient is formed by adjusting the flow rate. The delivered biomolecules can be changed over time within the scaffold, and the temporal modulation of these molecules can be achieved across the entire network in a spatially uniform manner [162]. These microfluidic devices were already successfully used to modulate neural tube patterning *in vitro*. Uzel et al. reported a microfluidic design to create orthogonal linear gradients in a 3D cell-embedded scaffold [163]. The authors used the reported device to generate gradients of retinoic acid (RA) and SAG, an agonist of the sonic hedgehog (SHH) [164], across a 3D collagen hydrogel with mouse ESC-derived aggregates [163]. Since RA has a caudalizing effect on the neuroepithelium and SHH is secreted in the most ventral part of the neural tube [165, 166], a combinatorial effect of these two morphogens specifies progenitor cells into caudal and ventral identities leading to the subsequent formation of ventral spinal cord neurons [163]. A similar approach was also used by Demers et al. In addition to RA and SHH signaling, they introduced a BMP4 gradient in a microfluidic device capable of mimicking the dorsal patterning of the neuroepithelium [167]. During neural differentiation, dorsal-ventral identity is achieved by establishment of opposing gradients of SHH and BMP, whereas the orthogonal delivery of the RA gradient allows the generation of the rostral-caudal axis [167]. These two different studies demonstrated the ability to generate temporally controlled morphogen gradients that allow the spatial patterning in stem cell-derived 3D structures.

Thus, the use of microfluidics can provide a transorganoid morphogen gradient, along with the immobilization of biomolecules in the biomaterial for spatial control [168]. In fact, direct integration of biomolecules into the scaffold allows to manipulate cell attachment, migration, and fate, but when combined with a delivery vehicle, like micro/nanoparticles, the controlled release of biomolecules is possible, allowing the generation of spatial gradients [169]. Mahoney and Saltzman were the first to assemble cells with the controlled release of polymeric microparticles to develop tissues with programmable synthetic extracellular microenvironments [170]. This technology was later applied to promote the controlled release of morphogens within organoids [171]. Degradable PLGA microspheres, containing RA, were incorporated within ESC-derived aggregates to achieve a controlled morphogen presentation and cystic spheroid formation [171]. An efficient cell differentiation and morphogenesis by the generation of structures that resemble the early mouse embryos (E6.75), with an exterior visceral endoderm enveloping an epiblast-like layer, was demonstrated [171]. Moreover, the combination of microparticles that present different kinetic releases allows a controlled and sequential morphogen presentation and therefore predetermine the time course of delivery and accomplish an efficient induction [172]. These approaches represent a versatile tool to create morphogen gradients that provide an accurate spatiotemporal regulation, being capable of inducing the symmetry breaking necessary for correct organoid morphogenesis.

## 5. Scaling Up of Organoid Generation

Other parameters, beyond biochemical signals and physical properties of ECM, should be considered for organoid generation, including sufficient nutrient and oxygen supply. The organoid size increases with the complexity of the generated structures, and it can range from 200 *μ*m to 4 mm [14]. Larger organoids usually present diffusion limitations making it hard to mimic some developmental features [173]. Based on the physics of diffusion, cell density, and the lower range of reported metabolic consumption rates for oxygen, cerebral organoids can achieve a maximal diameter of 1.4 mm without presenting central necrosis [174]. However, the predicted diameter is based only on the low metabolic activity present in the organoids, since spontaneous neural activity is infrequent [174]. The use of a dynamic system, like a spinning bioreactor, is able to support organoid growth due to an efficient transport of nutrients and oxygen diffusion, allowing the maintenance of large-size organoids, with about 4 mm, that efficiently recapitulate the cerebral structure [3]. In fact, bioreactors have been largely applied to expand and differentiate PSCs toward mesodermal, endodermal, and ectodermal lineages, without structural cellular arrangement within the stem cell-derived 3D aggregates [175–179]. The protocols for organoid generation using bioreactors typically involve initial commitment in static conditions and further embedding of the organoids within a hydrogel, followed by transferring organoids to the bioreactor [3, 180–182]. This methodology limits the potential scale-up and the application of organoid culture in high-throughput processes for drug discovery and toxicology studies. Recently, a new platform was reported that allows capsule production through electrospraying using a Matrigel core, yielding robust capsules with microenvironmental support and organoid growth through the generation of an outer alginate shell that protects the cell-Matrigel core [183]. However, the generation of controlled size aggregates and further differentiation into well-organized organoids using bioreactors, in a continuous process, remains a challenge. Moreover, how the bioreactor design can affect the organization and morphogenesis of the organoid is still poorly understood.

## 6. Conclusions and Future Challenges

The powerful self-organization capacity of PSCs has been demonstrated thought the *in vitro* generation of different mini organ-like structures, only by providing some critical cues. Therefore, *in vitro* morphogenesis recapitulation can provide significant insights for regenerative medicine, disease modeling, and drug screening applications. However, uncontrolled organogenesis can produce nonconsistent organoid anatomy and variable cellular composition, in which some cell types, as well as functional features, may be missing. The application of engineering methodologies to instruct organoid organization allows the better mimicking of human morphogenesis. In this review, we focused on distinct bioengineering approaches to achieve high levels of cellular organization within PSC-derived organoids, by controlling the initial cell position, spatiotemporal cellular stage, and remodeling of generated tissue.

3D recapitulation of human tissues offers the opportunity to better understand the biological systems, being a necessary reliable analysis of the organoids, with full characterization of the structure and function. However, only few methodologies allow the identification of the phenotype and morphogenesis without destroying the 3D organization of an organoid, by using advanced microscopy approaches. For example, using a clearing method, the scattering of tissues can be reduced and the structure becomes more transparent, enhancing deep light penetration into the tissues and imaging of deep structures [184]. In fact, with light-sheet microscopy, a 3D image is generated by scanning plane-by-plane through the sample, allowing deeper visualization in tissues with high spatiotemporal resolution [185]. In addition to imaging techniques, robust methods to evaluate the functionality of the generated 3D structures should be developed. For instance, electrophysiology is used to characterize the function of cardiomyocytes and neurons, since they are electrically excitable. Nevertheless, these techniques only permit the use of single cells (patch clamp) and monolayers (microelectrode arrays). Some adaptations have been made to record physiological parameters in the 3D constructs. In order to evaluate the functionality of the generated neuronal network in an intact system, the patch clamp has been performed in organoid sections [139, 186]. However, better methodologies for assessing the functional properties of whole organoids are needed.

In addition to the described challenges in the assessment of function and structure of organoids, our ability to generate organoids from PSCs has also been subjected to some limitations [187]. Firstly, there is a need to produce a significant number of organoids to use in high-throughput applications, as well as larger organoids are required to better recapitulate the anatomy seen in human tissues. Secondly, since PSC-derived organoids tend to form tissues reminiscent of human embryonic development, there is also the need to enhance the functionality of the generated tissues in order to produce more mature organoids. Attempts to overcome these challenges have been made, and organoids still have great potential to be used in biological and therapeutic studies aimed at better understanding human development and disease manifestation and at providing critical insights about effective therapies for several disorders.

## Figures and Tables

**Table 1:** Bioengineering approaches to control cell organization into PSC-derived organoids.

Self-organization control	Self-assembly	Scaffold-free approaches	Hanging drop method		[73–77]
			V-bottomed and round-bottomed multiwell plates		
			Microwells		
		Scaffold for imposing external and internal architecture	Nanotopography	Electrospinning	[88]
				Electron beam	
				Selective etching	
				Nanoimprinting	
			3D printing	Nozzle	[93, 94, 96, 188]
				Laser	
				Inkjet	
		Manipulation of organoid assembly	DNA-programmed assembly of cells		[100, 101]
			3D bioprinting	Inkjet bioprinting	[102–110, 112, 113, 189]
				Microextrusion systems	
				Laser-based direct-write techniques	
	Self-patterning and self-morphogenesis	Spatiotemporal control of mechanical signals	Synthetic ECM	Adhesion peptides	[146–148]
				Peptide substrates	
				Combined hydrogels	
			DNA-directed assembly of shape-controlled units		[149]
			Light-mediated patterning		[150, 151]
			pH-mediated patterning		[152]
			Supramolecular “host-guest” interactions		[153]
			Enzymatic reaction-mediated patterning		[154]
		Spatiotemporal control of morphogen diffusion	Light-mediated patterning		[160, 161]
			Microfluidic systems		[163, 167]
			Micro/nanoparticles		[170–172]
